# Transcription factor compensation during mammary gland development in E2F knockout mice

**DOI:** 10.1371/journal.pone.0194937

**Published:** 2018-04-04

**Authors:** Briana To, Eran R. Andrechek

**Affiliations:** Department of Physiology, Michigan State University, East Lansing, MI, United States of America; University of Tennessee Health Science Center, UNITED STATES

## Abstract

The E2F transcription factors control key elements of development, including mammary gland branching morphogenesis, with several E2Fs playing essential roles. Additional prior data has demonstrated that loss of individual E2Fs can be compensated by other E2F family members, but this has not been tested in a mammary gland developmental context. Here we have explored the role of the E2Fs and their ability to functionally compensate for each other during mammary gland development. Using gene expression from terminal end buds and chromatin immunoprecipitation data for E2F1, E2F2 and E2F3, we noted both overlapping and unique mammary development genes regulated by each of the E2Fs. Based on our computational findings and the fact that E2Fs share a common binding motif, we hypothesized that E2F transcription factors would compensate for each other during mammary development and function. To test this hypothesis, we generated RNA from E2F1^-/-^, E2F2^-/-^ and E2F3^+/-^ mouse mammary glands. QRT-PCR on mammary glands during pregnancy demonstrated increases in E2F2 and E2F3a in the E2F1^-/-^ mice and an increase in E2F2 levels in E2F3^+/-^ mice. During lactation we noted that E2F3b transcript levels were increased in the E2F2^-/-^ mice. Given that E2Fs have previously been noted to have the most striking effects on development during puberty, we hypothesized that loss of individual E2Fs would be compensated for at that time. Double mutant mice were generated and compared with the single knockouts. Loss of both E2F1 and E2F2 revealed a more striking phenotype than either knockout alone, indicating that E2F2 was compensating for E2F1 loss. Interestingly, while E2F2 was not able to functionally compensate for E2F3^+/-^ during mammary outgrowth, increased E2F2 expression was observed in E2F3^+/-^ mammary glands during pregnancy day 14.5 and lactation day 5. Together, these findings illustrate the specificity of E2F family members to compensate during development of the mammary gland.

## Introduction

The mouse mammary gland is composed of an arborized epithelial network embedded within a fat pad. At puberty, the mammary epithelium rapidly expands from a rudimentary structure with few branches to form Terminal End Buds (TEBs) that drive epithelial growth into the fat pad. These large club-shaped structures have a leading edge composed of cap cells that rapidly proliferate. As the cells migrate back into the center of the TEB they undergo apoptosis, forming a hollow tube [1], while those on the periphery differentiate into the luminal and myoepithelial layers. Once the epithelial network completely fills the fat pad, the TEBs are lost and the gland becomes largely static with small estrous related alterations. Upon the initiation of pregnancy, there is rapid proliferation and differentiation with well-regulated transcriptional programs to generate a lactating mammary gland. After weaning of the pups, apoptosis and remodeling of the gland occurs to return the mammary ductal network to a state closely resembling the nulliparous gland. This change and return to a virgin like state is also reflected in transcriptional programs, readily seen through a principle component plot [2]. Transcriptional studies of genes differentially expressed between the TEB and the ducts revealed numerous drivers of mammary growth [3, 4]. A study using a shRNA knockdown approach in Mammary Stem Cells (MaSC) identified a series of novel genes that influence MaSC and their ability to function as mammary stem or progenitor cells [5]. These studies, along with other transcriptional studies of mammary development [6, 7] have defined many of the transcriptional programs involved with mammary development and function.

In our previous analysis of mammary gland development, we predicted a role for the E2F family of transcription factors in mammary gland development [8]. The E2F transcription factors are commonly thought to regulate cell cycle, proliferation and apoptosis [9]. Individual developmental roles have been established with the knockouts of E2F1[10], E2F2[11], E2F3[12], E2F4[13] and E2F5[14]. More specifically, the role of E2F1-3 in development have been examined in the setting of the retina and small intestine using tissue specific knockouts [15, 16]. Interestingly, these E2F1-3 tissue specific triple knockout demonstrated that E2F1-3 are not crucial for normal cell proliferation but are needed for cell survival. However, in the setting of mammary gland development, E2F1 and E2F3 appear to have a role in proliferation and differentiation as evident by the delay in mammary gland outgrowth and branching defects. A delay in mammary gland outgrowth and branching defects was not observed in E2F2^-/-^ mice [8]. Outgrowth in this study was examined at 4 and 8 weeks of development with effects observed at both timepoints. However, virgin adult glands were not significantly different. In addition, loss of one copy of E2F3 resulted in a slight delay in involution. However, this effect was not noted in the other activator E2F knockouts. No effects were noted during pregnancy or lactation for the knockout strains [8]. Importantly, the binding motif for individual E2Fs is not distinct [17], requiring other proximal regions [18], likely indicating that E2Fs function with other co-activators [19]. As such, the loss of individual E2Fs in mammary gland development may therefore be compensated by other E2F family members [20]. This may occur both in developmental contexts as well as in cancer [21]. However, the extent of compensation in the mammary gland has not previously been explored.

Here we have integrated the development transcriptional studies [6, 7] with TEB gene expression [4] and the shRNA screen for MaSC genes [5] to predict a strong role for E2F activity in mammary gland proliferation and differentiation. Given the ability of the E2Fs to compensate, we then noted altered expression of E2Fs in individual E2F knockout backgrounds at various stages of mammary development. Examining mice lacking multiple E2Fs, we noted mammary gland outgrowth effects that were more extensive than for individual knockouts alone, indicating that there was significant compensation occurring. Combining our bioinformatic predictions with the individual and double E2F knockouts demonstrates the role of E2F compensation in mammary gland development.

## Materials and methods

### Animals

All mice were bred and maintained according to guidelines and protocols approved by the Institutional Animal Care and Use Committee in Michigan State University. Euthanasia was performed as mandated and approved through CO2 followed by a secondary method including necropsy. E2F1^-/-^, E2F2^-/-^, and E2F3^+/-^ mice were interbred to generate double knockouts in the FVB background. For wholemount generation, the inguinal mammary gland was excised and stained with Harris Modified Hematoxylin. To quantify the mammary epithelial outgrowth, the distance from the nipple to the leading edge of the epithelium and the distance from the nipple to the midpoint of the thoracic lymph node was measured. For the control, the ratio of the distance of outgrowth and distance between the lymph node and nipple was calculated. The ratio was set to 100% and used as standard to compare with various knockouts.

### Computational

Genes for the shRNA screening experiment were obtained from a public dataset [5]. The pooled shRNA screen was done by infecting MaSC-enriched basal cells with a customized mouse lentiviral library consisting of 1,296 shRNAmirs. The study identified potential regulators of MaSCs by observing altered mammosphere growth in the non-adherent mammosphere formation assay. TEB / duct genes were extracted from published data [4]. The TEB and duct were isolated mechanically and enzymatically from microdissected mammary gland of pubertal Balb/C mice aged 5–6 weeks. E2F1, E2F2 and E2F3 gene expression data and binary regression signature methodology used to generate signatures was as previously described [9, 22–25]. ChIP-seq data for numerous transcription factors was downloaded from public data [26, 27]. E2F1, E2F2 and E2F3 ChIP-Seq and ChIP-chip data was obtained from public sources [17, 28–30]. E2F1 ChIP-chip analysis was done in MCF10A cell lines. E2F2 ChIP-chip analysis was done in T lymphocytes isolated from 4 week old C57B16:129SV mice. E2F3 ChIP-chip and ChIP-seq analysis was completed in HCT116 cells and mouse myoblast and myotubes.

Unsupervised hierarchical clustering was completed with Cluster 3.0 using Euclidean distance and complete linkage and was visualized in Java Treeview. Heatmaps were altered to a blue/red color scheme using Matlab to ensure red-green color-blind viewers could distinguish the heatmap colors.

Transcription factor predictions based on motifs were generated using GATHER [31] and included the Bayes factor for statistical tests of enrichment. Other statistical tests were run using GraphPad Prism software and included Fishers two-tailed and t-tests.

### QRT-PCR

Mammary glands from three WT FVB, three E2F1^-/-^, three E2F2^-/-^ and three E2F3^+/-^ were excised on pregnancy day 14.5 and lactation day 5. Both of the number 4 inguinal mammary glands were collected from each mouse during necropsy. The mammary glands were snap frozen in liquid nitrogen and stored at -80°C. Pregnancy was confirmed through observation. For lactation samples, litters were standardized to 6 pups and glands were excised 4 hours after pups were removed. RNA was extracted from flash frozen mammary glands with the Qiagen RNeasy midi kit. Quantitative RT-PCR was performed using a SYBR Green One-Step RT-PCR Kit (Qiagen). The following primers were used (5’ to 3’): E2f1 forward, CGATTCTGACGTGCTGCTCT and reverse, CAGCGAGGTACTGATGGTCA; E2f2 forward, GCGCATCTATGACATCACCA and reverse, CGGGTGGGGTCTTCAAATAG; E2f3a forward, CCAGCAGCCTCTACACCAC and reverse, GGTACTGATGGCCACTCTCG; E2f3b forward, CTTTCGGAAATGCCCTTACA and reverse, GGTACTGATGGCCACTCTCG; Gapdh forward, TCATGACCACAGTGGATGCC and reverse, GGAGTTGCTGTTGAAGTCGC. Relative change was calculated using the ΔΔCt method. Statistical analysis was performed using an unpaired T-test.

## Results

**Fig 1:**
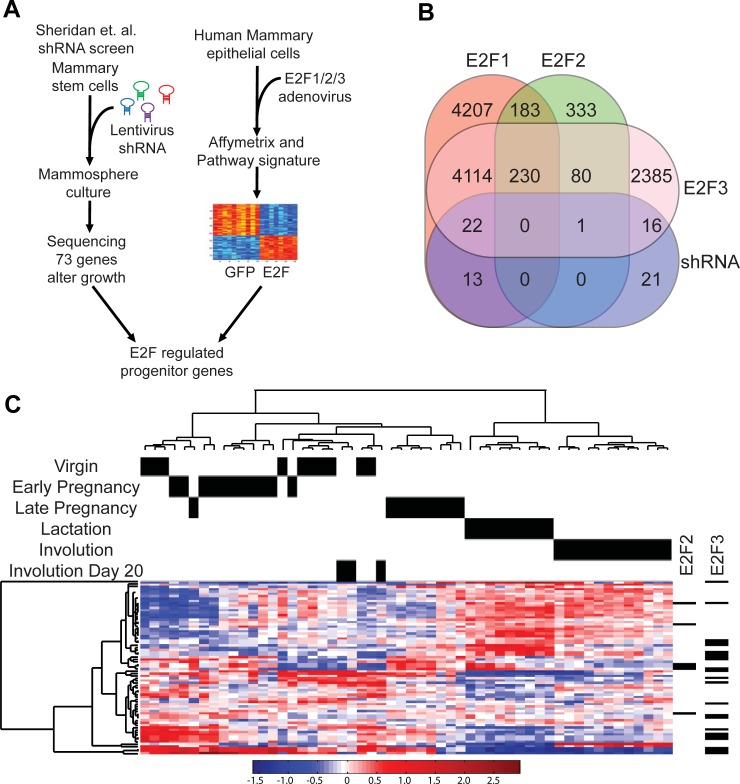
E2F transcription factors define mammary developmental states. Here we have combined a shRNA screen in primary mouse MaSC-enriched basal cells[5] with E2F1-3 gene expression signatures [25] to define progenitor genes regulated by the E2F transcription factors (A). Using a Venn diagram, overlap between E2F1, E2F2 and E2F3 target genes as determined by CHIP-seq/CHIP-chip and microarray experiments, and the 73 unique genes identified from the pooled shRNA screen was noted (B). Unsupervised hierarchical clustering of E2F1 signature genes from mammary glands collected at various stages of mammary development is stratified into the indicated stages. The overlap between E2F1, E2F2 and E2F3 signature genes is depicted on the right of the of the heatmap (C).

In order to explore the role of the E2F transcription factors in the regulation of mammary gland stem cells and progenitor cells, we have integrated the published shRNA screen data [5] with E2F signatures [23, 25] (Fig 1A). The shRNA screen was done in a CD29hi CD24+ subset of MaSC enriched basal cells which is a subpopulation of mammary cells that have the ability to reconstitute a complete mammary gland in vivo[5]. A customized mouse lentiviral library composed of 1,294 shRNAmirs targeted against genes involved in transcriptional regulation was used in the screen. The shRNA screen identified 73 genes that potentially regulate mammary stem and progenitor cell behavior, including genes with no previous implications in mammary gland development. The E2F signature data was derived from human mammary epithelial cells (HMECs) that had either GFP control adenovirus or E2F1, E2F2 or E2F3 adenovirus expression (23). Differentially expressed genes relative to the GFP control HMECs were identified for each of the activator E2Fs (Fig 1B). The genes in the various E2F signatures and in the shRNA screen for mammary stem and progenitor cells were then compared through a Venn diagram, revealing 34 shRNA screen genes that were potentially regulated by E2F1, 1 by E2F2 and 37 by E2F3. In addition, only 21 of the 73 potential stem and progenitor genes were not contained in an E2F signature dataset. Given the prevalence of E2F targets in the list of genes that regulate mammary stem and progenitor cells, we examined a mammary gland developmental gene expression dataset [6] for E2F1 targets. To accomplish this, we used the E2F1 signature genes [25], a subset of those shown in Fig 1B, defined through a binary regression approach. We clustered the mammary gland development dataset using only the genes present in the E2F1 signature, thus only genes that are regulated by E2F1 were used. Strikingly, this revealed that E2F1 target genes alone were able to cluster the various developmental phases assayed by gene expression (Fig 1C). This illustrates that E2F1 regulated genes are differentially expressed throughout the mammary developmental stages. Given the overlap in differentially regulated E2F genes (Fig 1B), we noted which genes were also contained in the E2F2 and E2F3 signatures (Fig 1C, right). Based on the small overlap between E2F2 targets and MaSC and progenitor cells (Fig 1B), it was not surprising that the E2F2 signature genes did not resolve the stages of mammary differentiation as clearly as E2F1 (S1A Fig). E2F3 signature genes also stratified mammary development stages in a manner similar to E2F1 (S1B Fig). To determine if a particular E2F is able to resolve the stages of mammary gland developments, we assessed how well late pregnancy, lactation and involution stages were able to be stratified.

**Fig 2:**
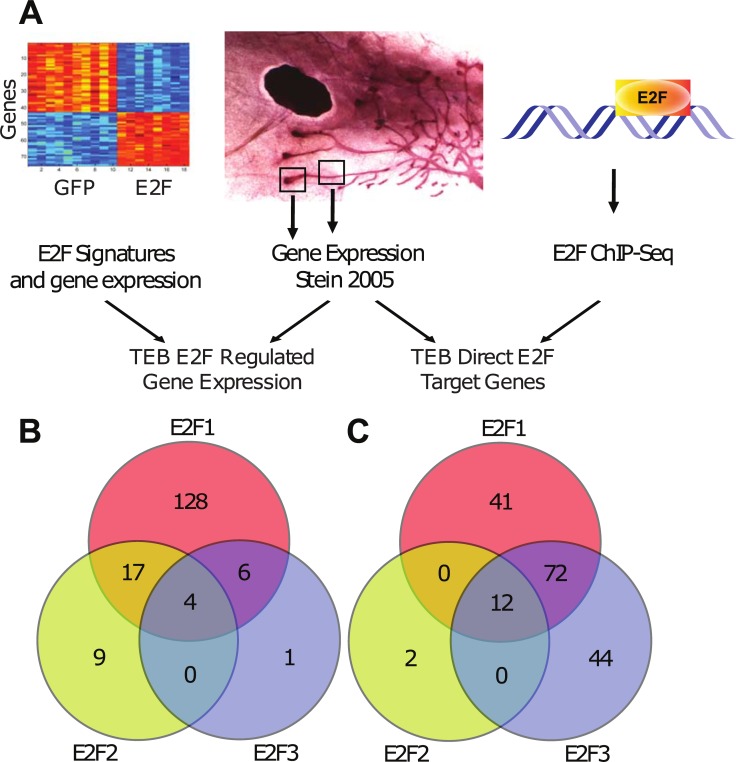
E2F regulated genes in the terminal end bud. Combining the E2F1, E2F2 and E2F3 signature genes and differentially expressed genes derived from microarray with a TEB / duct dataset [4] revealed E2F regulated genes in the TEB. Further, E2F ChIP-seq and ChIP-chip data was combined with this TEB/duct dataset to predict direct E2F targets in the TEB (A). Using Venn Diagrams to compare TEB E2F1, E2F2 and E2F3 signature genes/differentially expressed genes revealed which genes were regulated singly and by multiple E2F transcription factors (B). Similarly, direct TEB E2F1, E2F2 and E2F3 targets were analyzed (C).

With the noted roles for E2F1 and E2F3 in mammary stem cell and progenitor cells, we examined a TEB and duct gene expression dataset for E2F targets. Using a TEB vs duct gene expression dataset that was generated from microdissecting and enzymatic processing of TEBs and ducts (Fig 2A) [4], we identified a list of differentially expressed TEB genes with E2F gene enrichment. The TEB / duct gene list was compared to the gene expression from the E2F signatures (Fig 2B) and from E2F1, E2F2 and E2F3 ChIP-seq/ChIP-chip data (Fig 2C). Upon analysis of the genes that were shared between the differentially expressed TEB/duct genes and E2F signatures, we noted that E2F1 regulated genes were the most represented group (Fig 2B). These genes were equally split between being up regulated in the TEB and upregulated in ducts. In addition to key E2F signature genes, we also explored which direct targets from E2F ChIP-Seq/ChIP-chip experiments were represented in the TEB / duct dataset. This analysis clearly demonstrated that again E2F1 and E2F3 target genes were largely shared in the TEB and duct genes. In addition, we also noted 41 TEB genes that were direct targets of only E2F1 and 44 TEB genes that were direct targets of only E2F3. These two sets of differentially regulated E2F1 or E2F3 targets include genes that are involved in cell proliferation and division (S1 Table) This list includes genes that have previously described roles in mammary gland development including *Wnt5a*[32], *Cebpd*[33] and *Pttg1*. For example, deletion of *Pttg1*, which is regulated by E2F1, was reported to result in a defect in mammary gland branching and progression[34].

**Fig 3:**
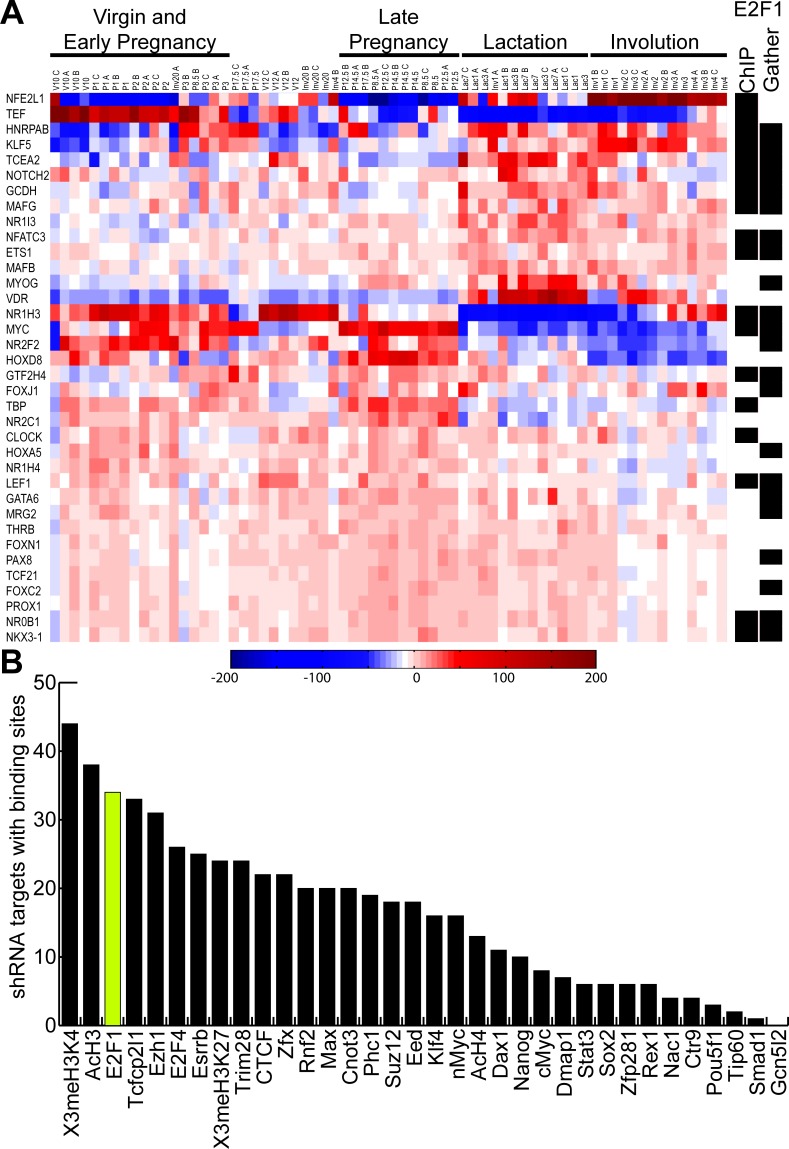
Progenitor genes, mammary stages and transcription factor enrichment. Unsupervised hierarchical clustering of 17 mammary gland developmental timepoints using genes with a potential role in mammary gland development as determined by the pooled shRNA screen. Each of the 17 developmental timepoints have three replicates. The overlap between potential mammary stem/progenitor cell genes and E2F1 direct and predicted target genes as determined by ChiP-ChIP and GATHER is depicted on the right side of the heatmap. The predicted E2F1 targets have a p-value of 0.002 and a Bayes factor of 3. (A). Examining transcription factors with overlap of ChIP data in the various stages of mammary development highlights the importance of these genes in mammary gland function. The E2F1 transcription factor is highlighted (yellow) with over 30 of the 73 genes being regulated (B).

In order to test the premise that the key mammary stem cell and progenitor genes were regulated by E2F transcription factors, we examined the expression of these genes throughout the mammary gland developmental cycle (Fig 3A). Filtering a mammary gland development dataset using only the list of 73 potential mammary stem cell and progenitor genes resulted in a list of 36 overlapping genes. Clustering based on these 36 genes alone stratified the mammary gland development gene dataset into various developmental stages. In addition, we demonstrated that many of these genes were either predicted (GATHER) or experimentally defined by ChIP-Seq/ChIP-chip to be E2F1 target genes. To further confirm E2F1 role in regulating mammary development, we performed a broad analysis of transcription factor binding (23) and isolated the genes that played a potential role in mammary stem cell development. This analysis demonstrated that amongst the group of various transcription factors, E2F1 was third in regulating the highest number of potential mammary stem cell genes (Fig 3B). Together, these findings along with the mammary development clustering data suggest that E2F1 and E2F3 are of critical importance to mammary gland development and function.

**Fig 4:**
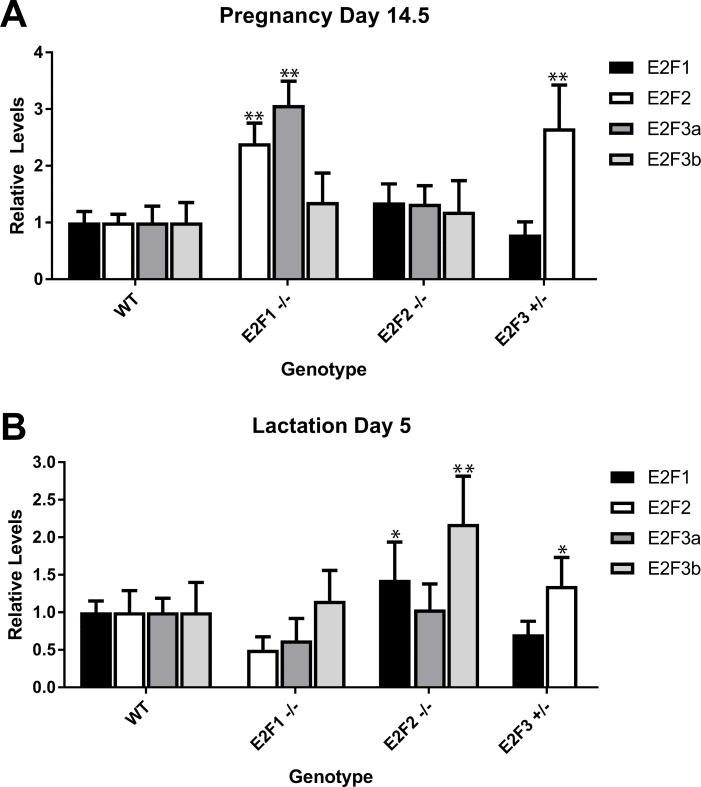
Compensation during mammary gland function. The levels of E2F1, E2F2, E2F3a and E2F3b were quantified in mammary gland RNA at pregnancy day 14.5 in wild type (WT) FVB (n = 3), E2F1^-/-^ (n = 3), E2F2^-/-^ (n = 3) and E2F3^+/-^ (n = 3) mice. A significant increase in E2F2 and E2F3a was observed in E2F1^-/-^ mice. A significant increase in E2F2 was also observed in E2F3^+/-^ mice (A). The levels of E2F1, E2F2, E2F3a and E2F3b were quantified at lactation day 5 in WT FVB (n = 3), E2F1^-/-^ (n = 3), E2F2^-/-^ (n = 3) and E2F3^+/-^ (n = 3) mice. A significant increase in E2F1 and E2F3b was observed in the E2F2 null mice. A significant increase in E2F2 was also noted in E2F3 heterozygous knockout mice (B). This analysis revealed differential E2F specific compensatory gene expression dependent upon the developmental context of the mammary gland. A significant increase in E2F expression levels relative to WT control mice are depicted with an asterisk. * p value < 0.05 and ** p value < 0.006.

In addition to the E2F transcription factors sharing a binding motif [18, 19], they are critical across all stages of mammary development (Fig 1) and are known to compensate for loss of other E2Fs [20, 21]. We therefore hypothesized that E2F transcription factors would compensate for each other during various stages of mammary development and function. Given that no major effects were previously noted [8] during pregnancy and lactation in E2F knockout mice, we hypothesized that the E2Fs were compensating for each other at this stage. To test this theory, mammary glands from control, E2F1^-/-^, E2F2^-/-^ and E2F3^+/-^ mice were collected and assayed by QRT-PCR for the other activator E2Fs. Relative to the wild type control, we observed a significant increase in E2F2 and E2F3a levels in the E2F1^-/-^ mice at pregnancy day 14.5 (p<0.05 for both). Similarly, E2F3^+/-^ mice had a large increase in only E2F2 levels during pregnancy (Fig 4A)(p<0.05). In addition to pregnancy, mammary glands from the 5^th^ day of lactation were examined. QRT-PCR at this timepoint demonstrated that there was a clear increase in E2F3b levels in the E2F2^-/-^ mice (Fig 4B)(p<0.05).

**Fig 5:**
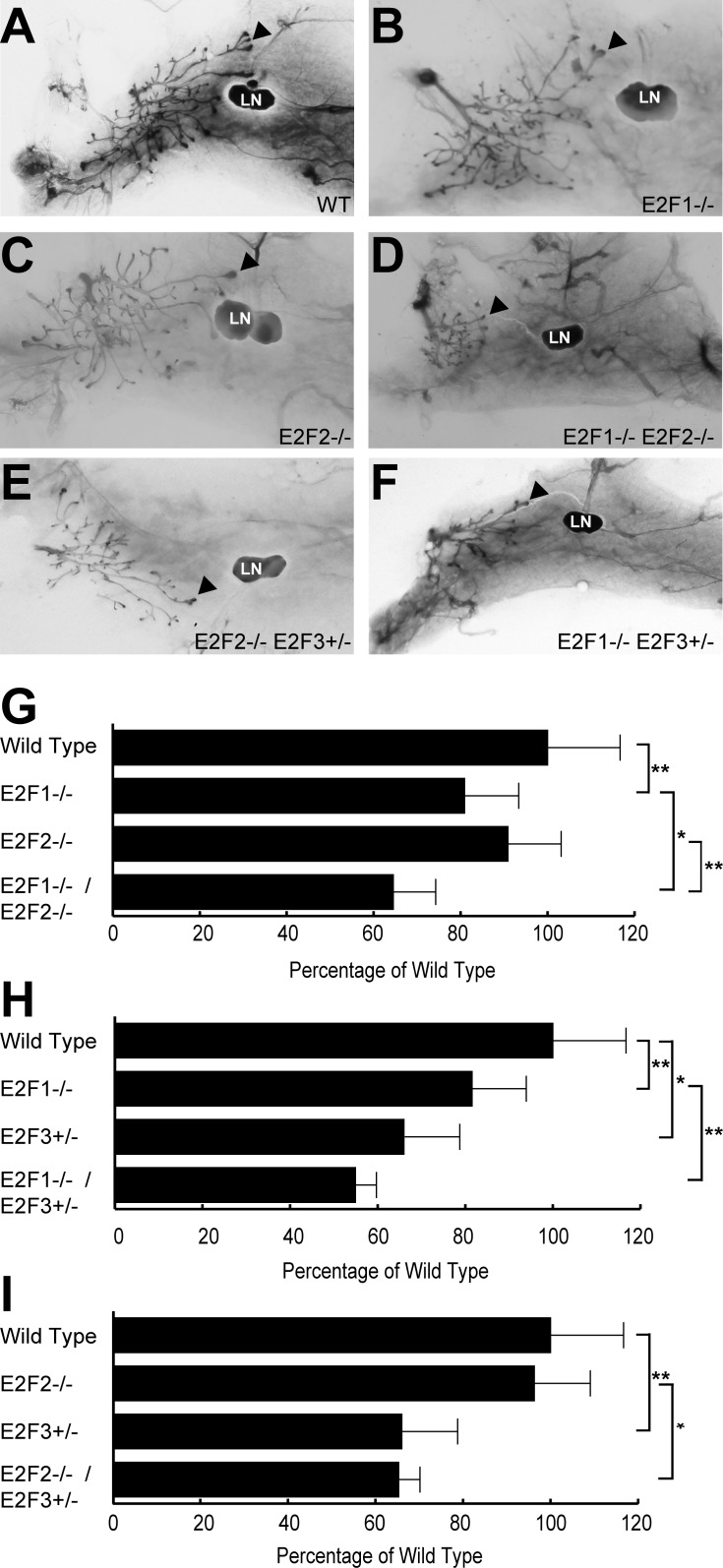
Mammary gland outgrowth in double knockouts. Representative wholemount images of mammary gland outgrowth at 4 weeks of development in wild type controls (A), E2F1^-/-^ (B), E2F2^-/-^ (C), E2F1^-/-^/E2F2^-/-^ (D) E2F2^-/-^/E2F3^+/-^ (E), E2F1^-/-^/E2F3^+/-^ (F) mice are shown. Measuring from nipple to lymph node (LN) as well as from nipple to the most distal TEB (arrowhead) allowed for quantification of mammary gland outgrowth. The results of the E2F1/E2F2 (E), E2F1/E2F3 (F) and E2F2/E2F3 (G) mammary outgrowth experiments are shown as a percentage of the wild type control growth. The mammary outgrowth was quantified in 12 WT FVB, 11 E2F1^-/-^, 15 E2F2^-/-^, 8 E2F3^+/-^, 5 E2F1^-/-^/E2F2^-/-^, 5 E2F1^-/-^/E2F3^+/-^ and 3 E2F2^-/-^/E2F3^+/-^. There is a significantly greater delay in mammary outgrowth in the E2F1^-/-^/E2F2^-/-^ mice relative to the E2F1^-/-^ mice. Differences in mammary outgrowth delay that are significant between two strains are depicted with an asterisk. * p value < 0.05 and ** p value < 0.006.

In a prior study of E2F function during development, it was noted that loss of individual E2Fs delayed mammary gland outgrowth [8] thus we hypothesized that the absence of multiple E2Fs would lead to greater outgrowth defect due to the disruption of a potential compensatory mechanism. In order to directly test the premise that E2Fs functionally could compensate for each other we interbred E2F1^-/-^, E2F2^-/-^ and E2F3^+/-^ mice to generate double knockout strains. Wholemounts of the mammary glands (Fig 5A–5D) revealed intriguing findings for each of the double knockout mice. Relative to wild type control, E2F1^-/-^/E2F2^-/-^ mice had a 40% delay in outgrowth (Fig 5E). This is a significantly greater delay in mammary outgrowth in comparison to the 20% reduction delay seen in E2F1^-/-^ mice and lack of delay in E2F2^-/-^ mice. These data indicate that E2F1 and E2F2 compensate for each other, resulting in a dramatic effect on the outgrowth of the double knockouts (Fig 5E). The E2F1^-/-^/ E2F3^+/-^ mice had a mild additive effect (Fig 5F), despite sharing many potential targets (Fig 2). Finally, the E2F2 ^-/-^/E2F3^+/-^ mice demonstrated that E2F2 was not able to compensate for loss of a copy of E2F3, with E2F3^+/-^ mice having the same outgrowth as the E2F2^-/-^/E2F3^+/-^ mice (Fig 5G). In all of the double knockout crosses, the mammary epithelial network was fully formed at 16 weeks of age, indicating that the outgrowth was only delayed. Taken together, these data indicate the specificity of the compensatory mechanisms in place for the E2F transcription factor family.

## Discussion

Here we have integrated shRNA screen data, gene expression signatures, developmental gene expression and ChIP-chip/ChIP-seq data to predict an overlapping role for E2F transcription factors in mammary gland development and function. This prediction was validated at a gene expression level in knockout mice during pregnancy and lactation and was experimentally tested in double knockout mice. The double knockout mice revealed specificity in the ability of E2Fs to compensate for loss of other family members. Together this integrative study underscores the importance of combining multiple large scale datasets to pose experimental questions.

The various bioinformatic analyses presented here strongly illustrates the importance of the E2F pathway in regulation of mammary gland development and function. This concept is reinforced by examination of the stratification of mammary gland developmental phases by genes from the E2F1 signature alone (Fig 1C). These effects are mediated through the regulation of genes within the TEB as well as later in functional states including lactation and involution. While E2F transcription factors are essential in this process, other transcription factors were also noted to potentially regulate the mammary stem cell and progenitor genes (Fig 3B). Interestingly, many of these promoters were noted to potentially regulate E2F activity through transcriptional repression and/or directly inhibiting its activity. This included transcription factors like Trim28 (Kap1), which has previously been shown to transcriptionally repress E2F1 and can also directly bind and inhibit its activity [35]. CTCF was identified in this assay and also has potential E2F co-factor activity [36]. Rnf2 was noted to regulate E2F1 activity through transcriptional repression after binding E2F promoters [37]. Together, these data illustrate the importance of the E2F transcription factors in the mammary stem cells.

The role of E2Fs in mammary gland development has been observed in our prior work [8], but the integration of various datasets indicated the potential for compensatory activity based on shared targets. While E2F compensation has been previously reported in both a tissue culture setting [20] as well as in our tumor models [21], the ability for E2Fs to compensate during mammary developmental processes has not previously been examined. Additionally, the specificity of the collaborative potential by individual E2Fs has not been explored. Based on the overlap of ChIP-seq/ChIP-chip data, E2F1 and E2F3 appeared to have significant potential for overlap. Interestingly, the loss of E2F1 resulted in an increase in both E2F2 and E2F3a expression during pregnancy. Examining the TEB genes that are direct targets of E2Fs also revealed shared genes between E2F1 and E2F3. Despite this, E2F1^-/-^ and E2F3^+/-^ mice both demonstrated delay in mammary outgrowth suggesting incomplete compensation. Given that mammary outgrowth is controlled by epithelial cells in the TEB, we believe that the TEB genes that are direct targets of either E2F1 or E2F3 are potentially responsible for the incomplete compensation during ductal elongation (Fig 2C). Furthermore, this list of differentially regulated E2F1 or E2F3 targets include genes involved in cell proliferation and division. Future work needs to be done to functionally test if these differentially regulated genes are responsible for the incomplete compensation. In addition, we identified a list of shared E2F1, E2F2 and E2F3 TEB targets that are potentially responsible for ductal development. However, whether knockout of these specific targets genes will functionally disrupt the compensation activity seen in the mammary gland of E2F individual knockout mice require further investigation.

Moreover, in the individual knockout experiments, E2F2 loss normally had no effect on mammary outgrowth, but the dual loss of E2F1 and E2F2 revealed that E2F2 was partially compensating for the loss of E2F1 since the delay was more profound in the double knockout. However, E2F2 was not able to compensate for the reduction in function of E2F3. Together these data illustrate the nature of the specificity in the ability of the E2F family members to compensate in mammary development and ductal morphogenesis.

The study of development is important to understanding other conditions, including cancer biology. In considering tumor biology, we have previously generated E2F knockout strains in MMTV-PyMT [38], MMTV-Neu [21] and MMTV-Myc transgenic mice [39]. In these experiments, we frequently noted altered gene expression of both direct and indirect E2F target genes. Additionally, increased metastasis with the loss of E2F2 in Myc induced tumors [40], and decreased metastasis with loss of E2F1 or E2F2 in Neu and PyMT induced tumors was observed. Consistent with these findings, E2F1 and E2F3 specific TEB targets includes genes that were previously reported to be involved with mammary tumor progression and metastasis in mice. Examples of these genes include *Loxl2*[41], *Klf4*[42], *Pdgfr*[43] and *Atf3*[44]. Importantly, previous studies have shown that elevated expression of upregulated genes in the E2F1 and E2F2 signature genes are associated with decreased time to distant metastasis free survival in breast cancer patients compared to those with low expression of these genes[21, 38, 40]. Based on these observations, we believe the biological relevance of the compensatory mechanism of E2Fs need to be carefully studied to understand their potential role in cancer biology.

## Supporting information

S1 Fig Unsupervised hierarchal clustering of mammary gland developmental expression. Unsupervised hierarchal clustering of a mammary gland developmental expression dataset using E2F2 signature genes stratified lactation and involution stages but not other stages of development (A). Unsupervised hierarchal clustering of a mammary gland developmental expression dataset using E2F3 signature genes was able to stratify early pregnancy, lactation and involution stages (B).
(EPS)

S1 Table E2F1 and E2F3 differentially regulated TEB genes. This table lists the TEB genes that are differentially regulated by E2F1 or E2F3 as determined by ChIP-seq/ChIP-chip analysis. This list of targets includes genes involved in cell proliferation and division.
(PDF)
