# Supplementary material for: Transcription factor compensation during mammary gland development in E2F knockout mice
Source: PLoS One. 2018 Apr 4;13(4):e0194937. doi: 10.1371/journal.pone.0194937 (PMC5884531; doi:10.1371/journal.pone.0194937)
Supplement: S1 Table — This table lists the TEB genes that are differentially regulated by E2F1 or E2F3 as determined by ChIP-seq/ChIP-chip analysis. This list of targets includes genes involved in cell proliferation and division. (PDF) [file pone.0194937.s002.pdf]

Table S1

**TEB E2F1 direct targets**	**TEB E2F3 direct targets**
CXCR7	PTGER3
SFRP2	IQGAP3
CST3	MKI67
PPAP2B	HNRNPA2B1
PLA2G7	NPNT
RASA4	WNT5A
RASD1	GOLIM4
DLL1	HMGB3
CTSB	GJB2
GEM	KCNN4
KRT17	PRSS23
NDN	GAS1
LOXL1	OSMR
COL6A1	FXYD2
FKBP7	RGS10
SGCE	COL5A1
IGFBP6	ACTA2
CTSL	TWIST1
COPS5	FGG
NSUN2	NFIL3
CKS1B	SERPINE1
C330027C09RIK	CCDC80
TAF9	COL1A1
TK1	PDGFRB
HSPA4	CEBPD
CLU	CXCL12
CDC20	RNASE4
PTTG1	ATF3
TACC3	PMP22
SMC4	COL6A2
KIF20A	BCL2A1C
ADAR	CX3CL1
AURKA	FOSB
SAT1	FHOD3
EMB	GAS6
MEST	CD83
KIF4	SMPDL3A
CCND1	AEBP1
TRIM17	COL15A1
FGB	COL5A2
CDCA8	FBN1
	ELN
	NRP1
